# Evaluating Patients' and Neonatologists' Satisfaction With the Use of Telemedicine for Neonatology Prenatal Consultations During the COVID-19 Pandemic

**DOI:** 10.3389/fped.2021.642369

**Published:** 2021-03-03

**Authors:** Maria C. Lapadula, Shanna Rolfs, Edgardo G. Szyld, Gene Hallford, Tracie Clark, Mike McCoy, Stephanie McKnight, Abhishek Makkar

**Affiliations:** ^1^Division of Newborn Medicine, Department of Pediatrics, University of Oklahoma Health Sciences Center, Oklahoma City, OK, United States; ^2^University of Oklahoma College of Medicine, Oklahoma City, OK, United States

**Keywords:** telemedecine, neonatology prenatal consultation, satisfaction survey, virtual prenatal visits, COVID-19 pandemic

## Abstract

**Background:** During the COVID-19 pandemic, telemedicine plays a critical role in providing safe, effective healthcare services, while reinforcing social distancing and optimizing the use of personal protective equipment. In this context, the Oklahoma Children's Hospital implemented virtual neonatology prenatal visits for pregnant women with a diagnosis of fetal anomalies. While tele-consultations have been broadly used with a high degree of acceptance in rural and remote areas, satisfaction has not been assessed in this particular scenario, where patients and physicians discussing sensitive healthcare information had to rapidly adjust to this new modality.

**Objectives:** To evaluate patients' and neonatologists' satisfaction with virtual prenatal consultations in the context of the COVID-19 pandemic and to compare satisfaction levels of patients receiving virtual consultation with those receiving in-person consults.

**Methods:** This cross-sectional study evaluated patients' and neonatologists' satisfaction with virtual consultations. Participants included pregnant women with diagnosis of fetal anomalies who received neonatology prenatal consultations at Oklahoma Children's Hospital, either in-person or through telemedicine, from May to mid-November 2020, and neonatologists providing virtual prenatal consultations in the same period. Virtual visits were delivered via Zoom Pro™. Patients and physicians who agreed to participate rated acceptability completing an anonymous 5-point Likert scale survey. Item frequencies and means for categories of items were computed by group (video-consult patients, in-person patients, physicians) and analyzed, using Welch's *t* for unequal sample size.

**Results:** Overall consultation quality was rated good or excellent by 35 (100%) video-consult patients and 12 (100%) in-person patients. Patient group means computed on six 5-point Likert items about patient-physician communication did not differ significantly, video-consult: *M* = 28.71 (2.22); in-person consult: *M* = 28.92 (1.78) (*p* = 0.753263). All eight physicians (100%) agreed or strongly agreed that telemedicine was effective, using a 5-point Likert scale, and their combined consultation quality score computed on 10 survey questions was high: *M* = 46.4 (3.11).

**Conclusion:** Despite patient inexperience with tele-consultations, the quick implementation of telemedicine, and the sensitive reason for the visit, patients and physicians were highly satisfied with virtual visits. Telemedicine is a safe, effective alternative for providing neonatology prenatal consultations for pregnant women with diagnosis of fetal anomalies during the pandemic.

## Introduction

The use of telecommunication technologies for medical purposes in the US date to the late 1950s (1). In the last three decades, with the proliferation of personal computers in the 1990s and more recently with smartphones, telemedicine became more popular. Research has found a high degree of acceptance among patients and providers, especially in rural and remote areas where access to specialists is limited (2–5). Virtual consultations have already been applied across different medical fields and have been shown to be effective and safe when used in appropriate clinical scenarios (6, 7).

The emergence of the novel coronavirus pandemic led to rapid and substantial changes in the way ambulatory care is delivered. The scope of telehealth abruptly grew worldwide (8) as a strategy for preventing patients' and providers' viral exposure and preserving personal protective equipment (PPE) while continuing to provide outpatient services. In April 2020, 43.5% of Medicare primary care visits were performed via telemedicine, compared with 0.1% reported in February same year, before the arrival of the COVID-19 pandemic (9). With unprecedented rapidity, all medical specialties adopted telehealth services, which have been shown to be a safe and effective solution to assure continuity of outpatient care, in a massive transition from in-person to virtual visits (10).

**Figure 1:**
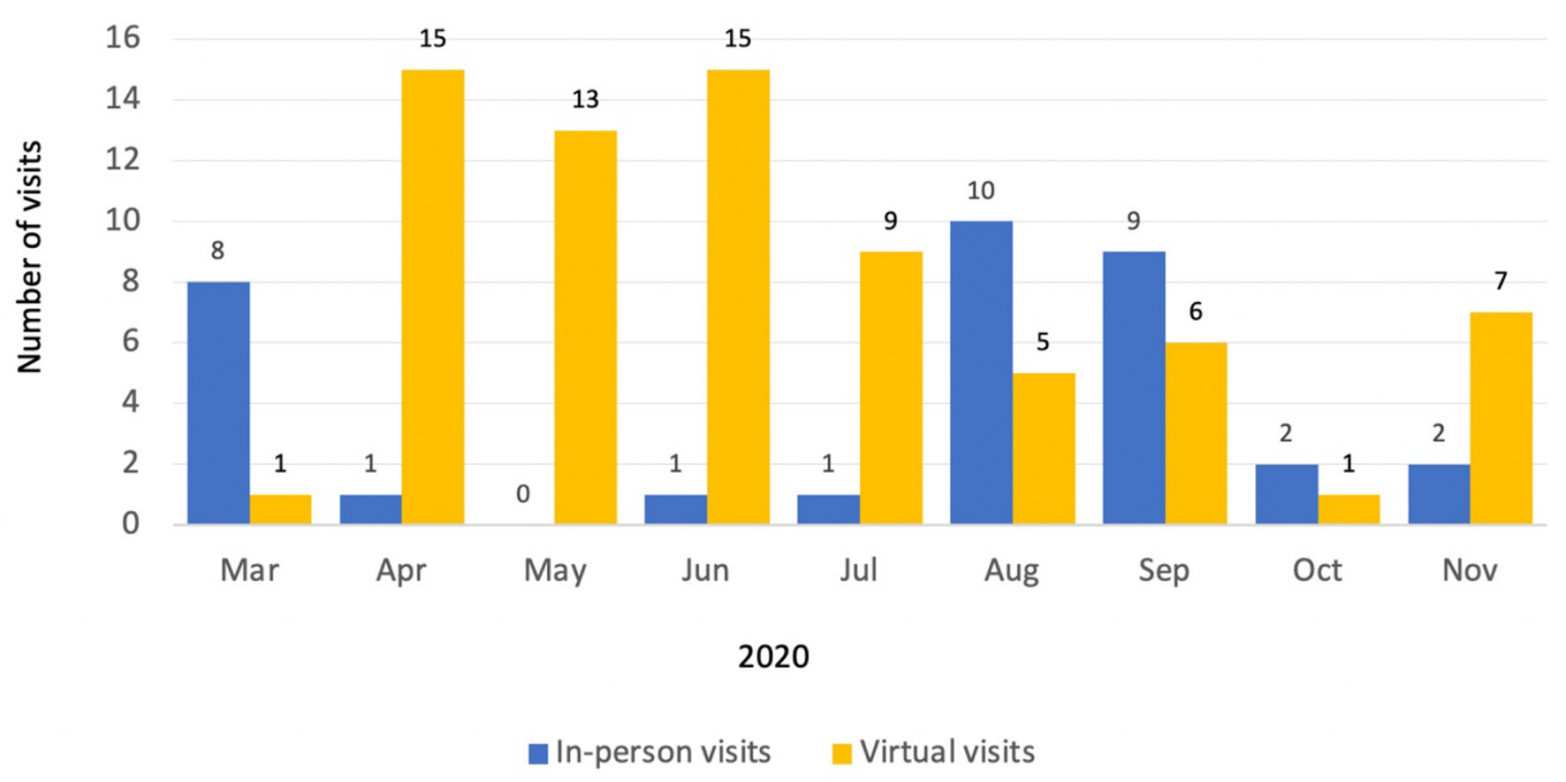
Neonatology prenatal consultations provided at Oklahoma Children's Hospital from March to mid-November 2020.

On these bases, the Section of Neonatal and Perinatal Medicine at Oklahoma Children's Hospital in Oklahoma City initiated virtual neonatology prenatal consultations for pregnant women with diagnosis of fetal anomalies, beginning in March 2020 (Figure 1). In ideal conditions, establishing a valid patient-physician relationship before the provision of telemedicine services is recommended (11). However, the current circumstances make this difficult. The transition to teleconsultations has been particularly challenging for neonatologists since the prenatal consult is typically the first encounter with these patients, and the reason for the visit is to communicate the diagnosis and prognosis of a congenital anomaly.

Delivering bad news is one of a physician's hardest tasks, and empathy and communication skills are essential (12). Since this was our first experience using telemedicine for these complex and sensitive consults and satisfaction has not been addressed in this context, we developed a survey to explore acceptability among users and providers. The purpose of this study was to assess patients' and neonatologists' satisfaction with virtual prenatal consultations during the COVID-19 pandemic.

## Methods

This cross-sectional study assessed satisfaction levels of patients and physicians using telemedicine for prenatal consultations during the COVID-19 pandemic in the Prenatal Diagnostic Clinic (PDC) at Oklahoma Children's Hospital in Oklahoma City. Prior to the project initiation, our research team submitted a plan of study to the Institutional Review Board (IRB) at the University of Oklahoma Medical Center. It was approved and granted a waiver of informed consent (IRB#12187), since no personal identifiers or medical information were to be collected as part of the study. Participation of both patients and professionals was anonymous and voluntary.

Pregnant patients with prenatally diagnosed fetal anomalies and who were seen at the PDC were offered and provided prenatal consultations by neonatologists concerning their baby's diagnosis and anticipated plan of care. Due to the COVID-19 pandemic, neonatologists had the option to provide the visits virtually, using the Zoom Pro™ platform, beginning in March 2020. Physicians received online training on how to use the platform before providing the service. In addition to familiarizing themselves with technology use, physicians were educated about specific requirements with virtual consultations, such as including verbal consent and platform used for the consultation in documentation and using modifier codes for billing. Of the 26 neonatologists within the Section of Neonatal and Perinatal Medicine, eight provided prenatal outpatient consultation service during the study period, having the majority of them performed both virtual and in-person visits. The decision whether or not to participate in virtual, was a matter of individual preference. Although we coordinated efforts for patients to be evaluated by the whole multidisciplinary team on the day they had their regularly-scheduled ultrasound, other specialists (neurosurgeons, obstetricians, pediatric cardiologists, etc.) provided consultation in person at the PDC as they have not implemented tele-consultations at the moment.

### Inclusion Criteria

The patient population included pregnant women with fetal anomalies who received prenatal visits with a neonatologist at Oklahoma Children's Hospital from May 1st to November 15th of 2020. Participating providers included neonatologists who delivered virtual prenatal consultations in the same time frame. Patients who received in-person consults were considered eligible to participate as a control group.

### Virtual Consultation Procedure

Tele-consultations were provided in a dedicated room ensuring patients' privacy and confidentiality via Zoom Pro™. After a patient's regularly-scheduled prenatal ultrasonography imaging, the patient was guided to the consultation room where she and the accompanying spouse or guest were requested to wait. At the time of the consultation, the PDC nurse navigator initiated a Zoom session with the consulting neonatologist and introduced the patient to her/him. Once the communication was established, the nurse navigator provided patients with a plain white envelope containing the anonymous satisfaction survey (Supplementary Figure A). Those willing to participate were asked to place the completed survey in a sealed envelope and leave it in a designated drop-box inside the room. At this time, the nurse navigator exited the room. Upon beginning the consultation, the neonatologist verbally consented for the virtual visit via Zoom. At the end of the consultation, the consulting physician notified the nurse navigator via text message that the appointment was over and instructed the patient to return to the waiting room. Then, the PDC nurse navigator wiped down the room and the computer and let the clinic staff know that the patient had returned to the waiting room.

In a similar fashion, patients receiving in-person consultations were given a questionnaire (Supplementary Figure B), identical to the one offered to virtual patients but without the questions relating to the telemedicine equipment and experience. This survey was provided to them in a plain white envelope and they were given the opportunity to complete the form and leave it in a designated drop-box in the consultation room. Physicians who provided virtual consultation during the study period received a survey (Supplementary Figure C) to be voluntarily filled and placed in a designated drop box.

All patients who received outpatient consultation got to meet their baby's neonatologist as inpatient once they were admitted to the hospital before delivery. At that time, the plan of care that was given to them during their outpatient prenatal consultation visit was reviewed and discussed.

### Satisfaction Survey

To address patients' satisfaction, we developed a survey using 5-point Likert scale items addressing perceived quality of care, physician's professional and communication skills, and the technology involved in the consultation. Physicians' satisfaction was measured on 5-point Likert scale items assessing previous experience with telemedicine, overall perception of the quality of healthcare provided, and technical aspects of the communication. Due to the anonymous nature of our survey and the small sample involved, we were not able to identify those physicians, if any, who solely provided virtual consultations or determine the overall demographic characteristics of the physician sample as a whole. We collected surveys from patients and providers involved in virtual visits from May 1st to November 15th of 2020. We obtained surveys from a convenience sample of patients who received in-person prenatal visits in the same time frame. Although no questions were asked about participants' ethnicity, a Spanish language version of the survey, translated by a certified translator, was available.

### Statistical Analysis

Frequencies and percentages were computed on all responses from virtual and in-person patients and providers. A composite consultation quality (CQ) score was created by summing participant responses from questions related to patient-physician communication. The CQ score included the six questions common to both the virtual and in-person surveys (Questions 2, 3, 4, 5, 6 and 7. See Supplementary Figure A). Each question had a possible range of 1 to 5, creating a possible composite score range of 1 to 30. A CQ score was also calculated for physicians; however, it was not statistically compared with the patients' CQ score as the physician survey consisted of different questions. The combined CQ score for physicians was calculated on ten 5-point Likert scale survey questions. Between-group comparisons were made on individual question responses and the composite CQ scores where appropriate, using Welch's *t* for unequal sample size.

## Results

### Participants

From May to mid-November, 81 patients received outpatient neonatology prenatal consultations. During that period, 50 patients completed and returned the satisfaction survey, being the overall response rate 61.7% (50/81). As the surveys were anonymous, we do not know the reasons why 31 patients decided not to participate. We speculate that after receiving unfavorable news, the willingness to participate in the study was understandably diminished. Of the 50 patients who completed the survey, 38 received virtual visits and 12 in-person visits. Three of the virtual patients' surveys were incomplete so only 35 were included in the final analysis. As this was a cross-sectional study using an anonymous questionnaire, participant demographic information was limited to age and level of education (Table 1). Eight consults were conducted in Spanish by a certified, Spanish-speaking physician. Four (11.43%) virtual consultation respondents and four in-person controls (33.33%) completed the survey in Spanish. English-speaking and Spanish-speaking participants were compared in both virtual and in-person patient groups on all survey questions and the composite CQ score. No statistically significant differences were found on any of these measures (Tables 2, 3).

**Table 1:** Patients' demographic variables.

**Parameter**	**In-person *N* = 12 *n* (%)**	**Telemedicine *N* = 35 n (%)**	***P*-value**
**Age in Years**			0.261547
Under 20	0	1 (2.86)	
20 to 39	10 (100)	32 (91.42)	
Over 40	0	1 (2.86)	
Did not answer	2	1 (2.86)	
**Education**			0.618918
Some school	4 (40)	4 (11.43)	
High school graduate	4 (40)	17 (48.57)	
Advanced education	2 (20)	12 (34.26)	
Did not answer	2	2 (5.71)	
**English Fluency**			0.171628
Yes	8 (66.67)	31 (88.57)	
No (Spanish-speakers)	4 (33.33)	4 (11.43)	

**Table 2:** Comparison of English-speaking vs. Spanish-speaking virtual patients (Welch's *t*-test for unequal N's).

**Comparison of English-speaking vs. Spanish-speaking virtual patients**				
	**English** ***N*** **=** **31**	**Spanish** ***N*** **=** **4**	***t*** **value**	***P-*****value**
Question 1	Mean = 4.65	Mean =4.75	0.389950	0.716434
Question 2	Mean = 4.65	Mean =4.50	−0.475708	0.660722
Question 3	Mean = 4.84	Mean = 4.75	−0.342693	0.751686
Question 4	Mean = 4.77	Mean = 4.75	−0.0925573	0.931196
Question 5	Mean = 4.867	Mean = 4.75	−0.452467	0.678318
Question 6	Mean = 4.84	Mean = 4.50	−1.142815	0.328521
Question 7	Mean = 4.84	Mean = 4.75	−0.342693	0.751686
Question 8	Mean = 4.84	Mean = 4.75	−0.321251	0.766393
Question 9	Mean = 4.77	Mean = 4.75	−0.0925573	0.931196
Question 10	Mean = 4.71	Mean = 4.50	−0.689967	0.531244
Question 11	Mean = 4.84	Mean = 4.50	−1.12907	0.330325
Satisfaction score	Mean = 28.81	Mean = 28.25	−0.373977	0.730217

**Table 3:** Comparison of English-speaking vs. Spanish-speaking in-person patients (Welch's *t*-test for unequal N's).

**Comparison of English-speaking vs. Spanish-speaking in-person patients**				
	**English** ***N*** **=** **8**	**Spanish** ***N*** **=** **4**	***T-*****value**	***P-*****value**
Question 2	Mean = 4.75	Mean =4.75	0.000000	1.000
Question 3	Mean = 4.88	Mean = 5.00	1.000001	0.350616
Question 4	Mean = 4.88	Mean = 5.00	1.000001	0.350616
Question 5	Mean = 4.75	Mean = 5.00	1.527525	0.170471
Question 6	Mean = 4.63	Mean = 4.50	−0.320061	0.757371
Question 7	Mean = 4.88	Mean = 5.00	1.000001	0.350616
Question 8	Mean = 4.88	Mean = 4.75	−0.447214	0.675132
Satisfaction score	Mean = 29.00	Mean = 29.75	0.941979	0.94197

Over the study period, eight neonatologists provided virtual prenatal consultations, and all completed and returned the anonymous survey. No demographic information was collected as the small sample size would have likely identified participants.

### Telemedicine-Related Issues

For 24 of the 35 patients who participated in tele-consultations, it was the first time they had received a virtual doctor's visit. All but one (97.14%) agreed or strongly agreed that they had been told in advance that the meeting with their doctor would take place through a videocall. One survey respondent was unsure whether or not they had been told before the visit. Thirty-four survey respondents (97.14%) reported feeling satisfied about talking with their baby's doctor through a videocall, with one survey completer reporting feeling neutral about the videocall.

All eight physicians agreed or strongly agreed that they had received adequate training in use of the telemedicine system for providing virtual neonatal visits (*N* = 8, 100%). All strongly agreed that the telemedicine system was both reliable and adequate for providing neonatal consults. Further, all agreed and strongly agreed that telemedicine is an effective way of delivering healthcare information to patients, and most agreed or strongly agreed that it is comparable in quality with in-person care (*N* = 7, 87.5%). The physician who felt neutral in that response explained in the comments' section that it would have been nice to be able to hand a tissue to the patient after communicating the news. All agreed or strongly agreed that they felt comfortable providing advice to patients *via* telemedicine, and all believed that it allowed for good patient interaction. As the primary reason for moving to virtual prenatal consults was due to the emergent COVID-19 pandemic, doctors were asked and all agreed or strongly agreed with feeling relieved delivering consults through telemedicine because it protected themselves and their patients from COVID-19 exposure. Finally, all eight neonatologists agreed or strongly agreed that their overall feeling about the use of telemedicine for prenatal consultations was good.

**Figure 2:**
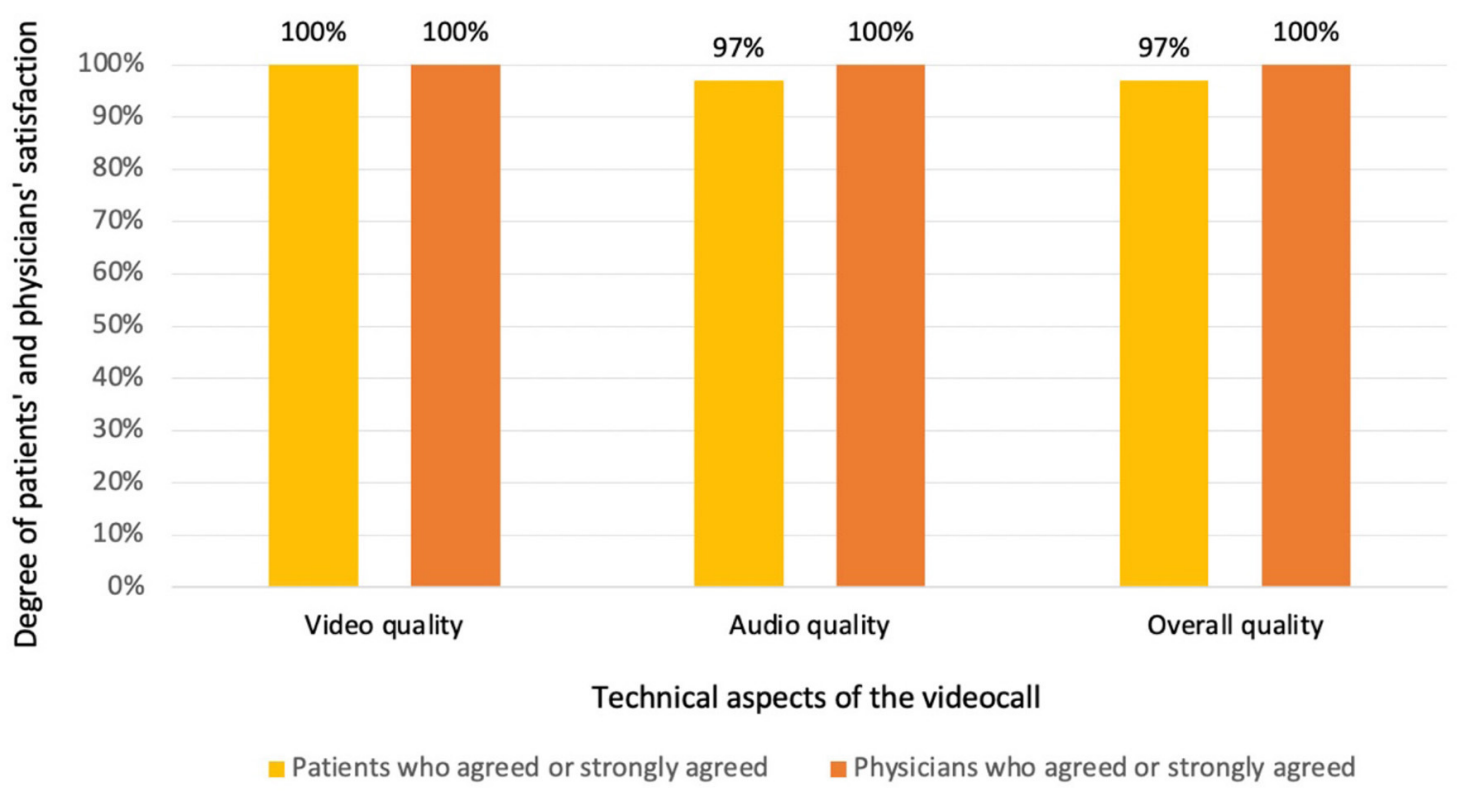
Patients' and physicians' satisfaction with the videocall quality.

When inquiring about the technological aspects of the communication, both patients and physicians reported a high level of satisfaction with the audio, video and overall quality of the videocall (Figure 2). Among survey respondents who answered the question, 98.7% indicated that they would participate in virtual doctor visits in the future.

### Prenatal Consultation Quality of Care Questions

**Figure 3:**
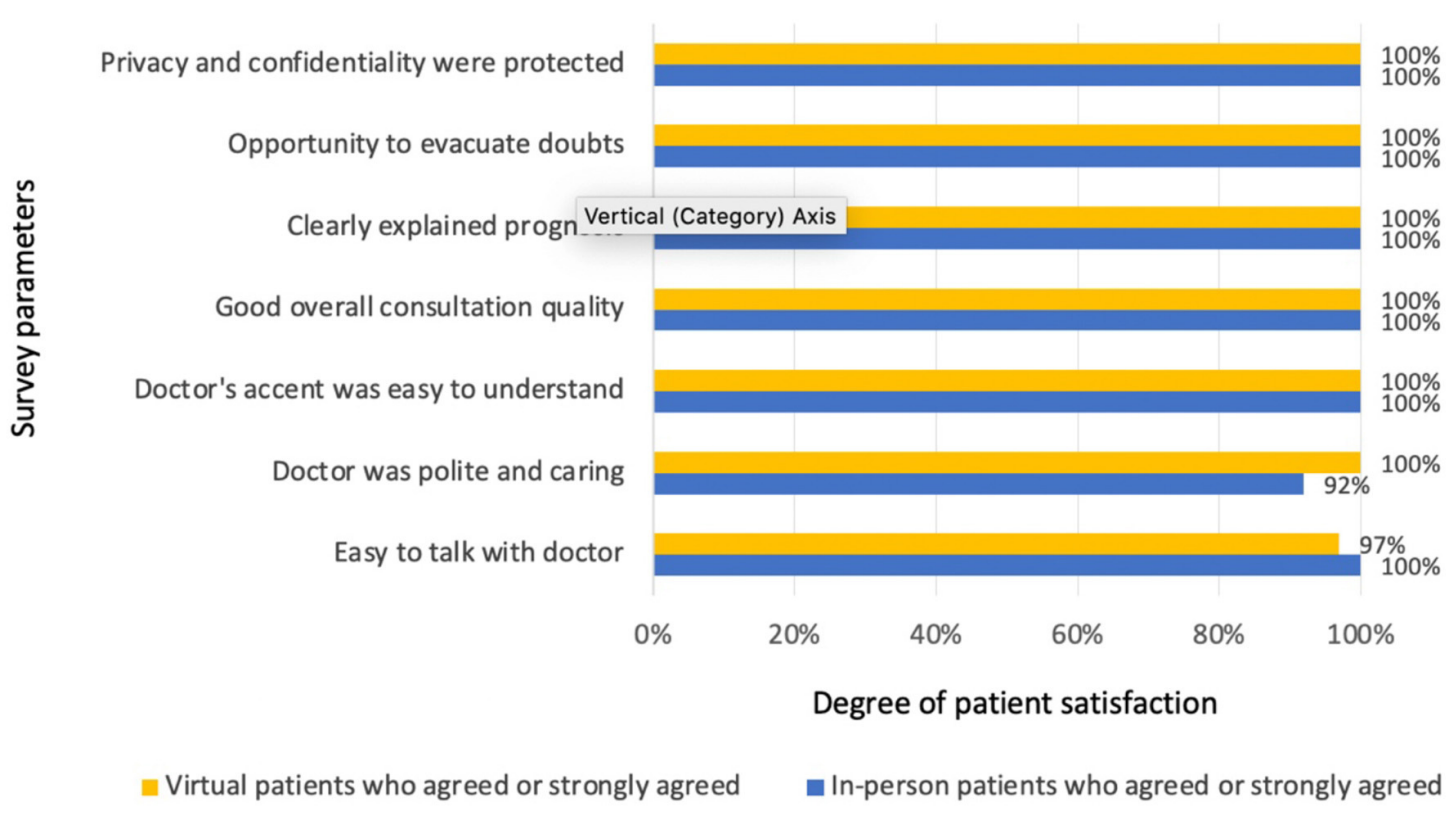
Patients' satisfaction with prenatal consultations in both groups (virtual and in-person visits).

The overall quality of the consultation, as well as satisfaction with individual components of the visit regarding physicians' professional and communicational skills, were highly rated among patients in both groups. The items included in the satisfaction survey and the percentage of patients who agreed and strongly agreed in their responses are represented in Figure 3. Virtual patients agreed or strongly agreed that it was easy to talk to their baby's doctor through the videocall (*N* = 34, 97.14%), while all (100%) control patients agreed or strongly agreed that it was easy to talk to their baby's doctor during the in-person visit. Similarly, all 35 virtual and 11 of 12 (91.68%) in-person patients agreed or strongly agreed that their baby's doctor was polite and caring. All virtual (*N* = 35) and in-person (*N* = 12) patients agreed or strongly agreed that their baby's doctor's accent was easy to understand.

### Consultation Quality Scores

Statistical comparison of virtual vs. in-person groups on the survey question rating overall consultation quality and the combined CQ score revealed no significant difference on either measure (Table 4). The combined CQ score for physicians showed a high mean across all items (*M* = 46.4 ± 3.11) (Table 5).

**Table 4:** Patients' perceptions of the overall consult quality and composite CQ score group means computed on six 5-point Likert items about patient-doctor communication.

	**Virtual (*N* = 35)**	**In-person (*N* = 12)**	***P*-value**
Overall consult quality	Mean = 4.83 SD = 0.38	Mean = 4.83 SD = 0.40	0.971083
Composite CQ score	Mean = 28.71 SD = 2.22	Mean = 28.92 SD = 1.78	0.753263

**Table 5:** Physicians' perceptions of the overall consult quality and composite CQ score group means computed on ten 5-point Likert scale survey questions.

	**Mean (SD)**	**Lowest**	**Highest**
Overall consult quality	4.63 (0.52)	4	5
Composite CQ score	46.38 (3.11)	42	50

## Discussion

After evaluating different components of satisfaction among patients and neonatologists with the use of telemedicine for prenatal consultations, we found a similar degree of fulfillment with virtual visits when compared with in-person consultation. Despite the rapid implementation of telemedicine and the limited training physicians received, virtual consultations met users' and providers' expectations. A reasonable interpretation of the results is that these consults do not require physical examination and the success depends on good patient-physician communication.

The ability to listen and empathize with patients while providing information in a lay language is important in any medical consultation (13), but is essential when delivering bad news. It was our concern that the use of telemedicine would make it more difficult for patients and physicians to understand each other due to differences in regional dialects and cultures. Patients receiving virtual visits, regardless of the language they spoke, agreed that talking to the doctor was easy, the accent was understandable and that they felt content (the doctor was polite and caring). Ironically, in pandemic days, non-verbal cues are easier to detect through a videocall where patients and physicians are allowed to remove their face masks than in “traditional” visits in which masks and a six-foot-distance are required.

Although all physicians agreed that telemedicine was a reliable, effective tool for providing prenatal consultations, one was not sure about its quality being equal to in-person visits, arguing that it would have been nice to be able to hand a tissue to the patient. That is not a minor comment; as virtual visits rise in number, so does the need to develop new techniques to emotionally support our patients through distance technologies (14).

Even though two thirds of virtual patients were participating in a tele-consultation for the first time, most were satisfied about talking to their baby's doctor through telemedicine and would participate in a virtual visit in the future. Based on these results, we are considering providing virtual visits to patients in their homes, respecting patients' times and reducing transport costs while allowing them to connect more frequently and easily. Bishop et al. recently demonstrated the feasibility of providing prenatal consultation where the patient was located at home, but they did not evaluate patient and provider satisfaction with telemedicine use (15).

To our knowledge, this is the first study showing the satisfaction component of prenatal consultations for patients with fetal anomalies. In accordance with previous studies conducted in related fields, telemedicine was shown to be a safe and effective tool with which to provide ambulatory consultations with a high satisfaction rate among users and providers (16–18).

Our study may have important economic implications. Although we did not calculate the reduction in healthcare costs, we optimized the use of PPE and reduced potential exposures of both patients and staff, since some neonatologists provided the visits from their homes. Also, telemedicine consultations during the pandemic have allowed us to efficiently manage our workforce, which was reduced due to staff illness and quarantine requirements.

Limitations include the small sample size, the fact that patients' acceptability of tele-consultations could have been influenced by the COVID-19 pandemic context, and that participating physicians had elected to conduct virtual visits. While physicians had the option to provide virtual or in-person visits, patients did not have the opportunity to choose. However, none refused to receive a virtual visit, and all reported a high-quality perception of the visit. Because of the anonymous nature of the survey, we did not attempt to link diagnosis with degree of satisfaction reported by patients. However, we do not believe that this variable influenced their responses as the overall parental satisfaction, either with virtual or in-person prenatal consultations, was very high.

Prior to the pandemic, telemedicine was mostly used in rural and remote areas for patients with limited access to specialized care (19, 20). With the outbreak of COVID-19, the flexibilization of federal and state regulations and reimbursement policies allowed for telemedicine expansion. We are experiencing an unprecedented shift in the way we deliver outpatient care. While face-to-face visits are the best-known model with which to provide healthcare and are irreplaceable in many cases, new technologies emerged and are changing the paradigm of outpatient care delivery. The pandemic brought the opportunity to implement telemedicine in almost all medical fields and was shown to be safe, efficient and cost-effective when appropriately used. Once the COVID-19 restrictions are released, video-consultations may be a suitable option for selected patients, reducing the need for face-to-face visits without decreasing healthcare quality or patient satisfaction. Additionally, offering a consultation with the whole multidisciplinary team in same room, either virtual or in-person, would result in a significant improvement in the quality of care provided to these patients.

In conclusion, after exploring satisfaction with the use of telemedicine for prenatal consultations during the current pandemic, we found a high degree of acceptance from both patients and neonatologists. When comparing satisfaction levels of patients receiving virtual visits with those receiving in-person visits, we found telemedicine to be non-inferior to traditional consultations in terms of perceived quality of care. Despite the rapid implementation of the new platform and the scarce training neonatologists received, they were able to establish a good patient-physician communication, clarify patients concerns, and convey empathy, which is particularly important in these sensitive consults. Based on our findings, we anticipate that telemedicine could be effectively used in the future to provide neonatology prenatal consultations to patients not only within the hospital, but also in their homes.

## Data Availability Statement

The raw data supporting the conclusions of this article will be made available by the authors, without undue reservation.

## Author Contributions

ML contributed to study design, data analysis and manuscript preparation. AM contributed to study design, data analysis and manuscript preparation. SR contributed to data collection and manuscript preparation. ES contributed to study design and manuscript preparation. GH contributed to data analysis and manuscript preparation. TC contributed to study design and manuscript preparation. MM contributed to data collection. SM contributed to data collection. All authors approved the final manuscript as submitted and agree to be accountable for all aspects of the work.

## Conflict of Interest

The authors declare that the research was conducted in the absence of any commercial or financial relationships that could be construed as a potential conflict of interest.

## Abbreviations

PPE: personal protective equipment
PDC: prenatal diagnostic clinic
IRB: institutional review board
CQ: consultation quality.
