# Supplementary material for: Evaluating Patients' and Neonatologists' Satisfaction With the Use of Telemedicine for Neonatology Prenatal Consultations During the COVID-19 Pandemic
Source: Front Pediatr. 2021 Mar 3;9:642369. doi: 10.3389/fped.2021.642369 (PMC7966517; doi:10.3389/fped.2021.642369)
Supplement: Supplementary file 1 [file Data_Sheet_1.PDF]

1. **Supplementary Material**

1.1 **Supplementary Figure A. Virtual patients' satisfaction survey.**

Telemedicine Parental Satisfaction Survey

We would like to know how you felt about your visit with your Baby's doctor, using a computer (videocall). Your answers will help us to make improvements to our program and to provide the highest quality care to you and your child.

Participation in this survey is anonymous and voluntary. Please do not write your name or your Baby's name, or any other information that could identify you (phone number, mailing address, etc.) on this survey. Once you begin, you may decide not to complete this survey. If you decide not to participate, you may either take the survey with you or return it as blank in the black survey box. If you decide to participate and complete the survey, please return it in the black survey box.

Is this the first time you have had a doctor's visit by videocall? YES ○ NO ○

**Statement**	**Strongly Agree**	**Agree**	**Neutral**	**Disagree**	**Strongly Disagree**
1. It was explained to me ahead of time that I would be meeting with my Baby's doctor through videocall.	○	○	○	○	○
2. It was easy to talk to my Baby's doctor through videocall.	○	○	○	○	○
3. My privacy and confidentiality were respected and protected during the consult.	○	○	○	○	○
4. The doctor explained to me clearly what to expect once my Baby is born.	○	○	○	○	○
5. I feel I had the opportunity to ask all my questions.	○	○	○	○	○
6. My Baby's doctor's accent was easy to understand.	○	○	○	○	○
7. My Baby's doctor was polite and caring.	○	○	○	○	○

**Statement**	**Excellent**	**Good**	**Fair**	**Poor**	**Very Poor**

8. The overall quality of the consultation I received was:	◯	◯	◯	◯	◯
**Statement**	**Excellent**	**Good**	**Fair**	**Poor**	**Very Poor**
9. The picture quality of the videocall was:	◯	◯	◯	◯	◯
10. The sound quality of the videocall was:	◯	◯	◯	◯	◯
**Statement**	**Very Satisfied**	**Moderatel y satisfied**	**Neutral**	**Modera-tely dissatis-fied**	**Very dissatis-fied**
11. My overall feeling about talking with my Baby's doctor through videocall was:	◯	◯	◯	◯	◯

What's your age range?

- ◯ Under 20
- ◯ 20-29
- ◯ 30-39
- ◯ 40-49
- ◯ 50 or more

What's your highest level of education?

- ◯ Some schooling but did not graduate
- ◯ High school graduate
- ◯ Associate degree
- ◯ Bachelor's degree
- ◯ Master's degree
- ◯ Professional degree

Would you be willing to use videocall to talk to the doctor in the future? YES ◯ NO ◯

Please write any other comments or suggestions here:

Thank you for your participation!
