# Supplementary material for: Evaluating Patients' and Neonatologists' Satisfaction With the Use of Telemedicine for Neonatology Prenatal Consultations During the COVID-19 Pandemic
Source: Front Pediatr. 2021 Mar 3;9:642369. doi: 10.3389/fped.2021.642369 (PMC7966517; doi:10.3389/fped.2021.642369)
Supplement: Supplementary file 2 [file Data_Sheet_2.PDF]

1. **Supplementary Material**

1.1 **Supplementary Figure B. In-person patient satisfaction survey.**

Prenatal Consultation Satisfaction Survey

We would like to know how you feel after visiting your Baby's doctor today. Your answers will help us to make improvements to our program and to provide the highest quality care to you and your child.

Participation in this survey is anonymous and voluntary. Please do not write your name or your Baby's name, or any other information that could identify you (phone number, mailing address, etc.) on this survey. Once you begin, you may decide not to complete this survey. If you decide not to participate, you may either take the survey with you or return it as blank in the black survey box. If you decide to participate and complete the survey, please return it in the black survey box.

Is this your first consultation with your Baby's doctor? YES ○ NO ○

Statement	Strongly Agree	Agree	Neutral	Disagree	Strongly Disagree
1. It was easy to talk to my Baby's doctor.	○	○	○	○	○
2. My privacy and confidentiality were respected and protected during the consult.	○	○	○	○	○
3. The doctor explained to me clearly what to expect once my Baby is born.	○	○	○	○	○
4. I feel I had the opportunity to ask all my questions.	○	○	○	○	○
5. My Baby's doctor's accent was easy to understand.	○	○	○	○	○
6. My Baby's doctor was polite and caring.	○	○	○	○	○

Statement	Excellent	Good	Fair	Poor	Very Poor

7. The overall quality of the consultation I received was: ○ ○ ○ ○ ○

What's your age range?

- Under 20
- 20-29
- 30-39
- 40-49
- 50 or more

What's your highest level of education?

- Some schooling but did not graduate
- High school graduate
- Associate degree
- Bachelor's degree
- Master's degree
- Professional degree

Please write any other comments or suggestions here:

Thank you for your participation!
