# Supplementary material for: Evaluating Patients' and Neonatologists' Satisfaction With the Use of Telemedicine for Neonatology Prenatal Consultations During the COVID-19 Pandemic
Source: Front Pediatr. 2021 Mar 3;9:642369. doi: 10.3389/fped.2021.642369 (PMC7966517; doi:10.3389/fped.2021.642369)
Supplement: Supplementary file 3 [file Data_Sheet_3.PDF]

1. Supplementary Material

1.1 Supplementary Figure C. Physicians' satisfaction survey.

Telemedicine Physician Satisfaction Survey

We would like to assess physicians' satisfaction with using telemedicine for patient encounters. Your answers will help us to make improvements to our program and to provide the highest quality care.

Participation in this survey is anonymous and voluntary. Please do not write your name or any other information that could identify you.

Have you conducted prenatal consultations in an outpatient setting in the last 12 months?

YES ○ NO ○

If your answer is YES please, continue with the survey

Statement	Strongly Agree	Agree	Neutral	Disagree	Strongly Disagree
1. I feel comfortable giving patient's advice using telemedicine.	○	○	○	○	○
2. I believe telemedicine visits are effective in delivering health care information to patients.	○	○	○	○	○
3. I believe that my patient's experience using telemedicine has been good.	○	○	○	○	○
4. Patient care through telemedicine is comparable in quality with in-person patient care.	○	○	○	○	○
5. The telemedicine system is both reliable and adequate for providing prenatal consultations.	○	○	○	○	○
6. In my experience, patients are able to interact with and ask questions during telemedicine sessions.	○	○	○	○	○
7. In my experience, patients are usually satisfied with the	○	○	○	○	○

care received through telemedicine.					
8. I felt comfortable assessing and planning for my patient care with telemedicine.	○	○	○	○	○
9. I felt relieved delivering consults trough telemedicine because I'm protecting my patients and myself from COVID-19 exposure.	○	○	○	○	○
**Statement**	**Strongly Agree**	**Agree**	**Neutral**	**Disagree**	**Strongly Disagree**
10. I received adequate training to use telemedicine for virtual neonatal patient visits.	○	○	○	○	○
11. My overall feeling about using telemedicine for virtual patient visits has been good.	○	○	○	○	○
**Statement**	**Excellent**	**Good**	**Fair**	**Poor**	**Very Poor**
12. The picture quality of the videocall was:	○	○	○	○	○
13. The sound quality of the videocall was:	○	○	○	○	○

Additional Questions:

Have you used telemedicine for any other application besides providing prenatal consultations?

YES ○ NO ○

If your answer is YES please, continue with the survey

How long have you been using telemedicine?

Less than 6 months ○ 6-12 months ○ More than 12 months ○

How many virtual patient visits/encounters have you conducted using telemedicine in the last year?

Less than 5 ○ 6-20 ○ 21-50 ○ More than 51 ○

Please write any other comments or suggestions here:

Thank you for your participation!
